# Finite Element and Theoretical Analysis of High-Strength Steel-Strand Mesh Reinforced ECCs Under Flexural Load

**DOI:** 10.3390/ma17235943

**Published:** 2024-12-04

**Authors:** Lei Cao, Ziyuan Li, Yuxuan Li, Ke Li, Denghu Jing, Ya Qi, Yaohui Geng

**Affiliations:** 1 School of Civil Engineering and Transportation Engineering, Yellow River Conservancy Technical Institute, Kaifeng 475000, China; 2 School of Civil Engineering, Zhengzhou University, Zhengzhou 450001, China; 3 School of Civil Engineering, Southwest Jiaotong University, Chengdu 611756, China; 4 School of Civil Engineering, Southeast University, Nanjing 211189, China

**Keywords:** engineered cementitious composites (ECCs), high-strength steel-strand mesh (HSSM), flexural performance, finite element analysis (FEA), bond–slip behavior

## Abstract

This research investigates the flexural performance of slabs reinforced with high-strength steel-strand mesh (HSSM) and engineered cementitious composites (ECCs). By employing finite element analysis (FEA) and theoretical modeling, this study aims to deepen the understanding of how these materials behave under bending stresses. A finite element model was developed to simulate the nonlinear behavior of ECCs during bending, considering critical elements such as tensile and compressive damage, as well as bond–slip interactions between the steel strands and the ECCs. Furthermore, a theoretical model was created to predict the load-bearing capacity of HSSM-reinforced ECC slabs, incorporating variables like reinforcement ratios, slab dimensions, and material characteristics. The findings reveal that increasing the reinforcement ratio substantially enhances both flexural stiffness and load-bearing capacity while reducing deflection. Comparisons between the FEA results, the theoretical forecasts, and the experimental observations show close alignment, validating the proposed models. This work provides important insights for optimizing the design of HSSM-reinforced ECC slabs, highlighting their potential improvements in structural systems that demand high flexural performance.

## 1. Introduction

Continuous advancements in civil engineering and material science have significantly driven the development of high-performance construction materials to meet the growing demands of modern infrastructure. Among these materials, reinforced concrete remains widely used due to its availability and ease of application. However, it suffers from inherent drawbacks such as brittleness and low tensile strength, which hinder its performance in demanding environments requiring durability and resilience. The limited tensile strain capacity of reinforced concrete predisposes it to large-scale cracking or catastrophic failure when subjected to dynamic loads or harsh environmental conditions [1,2,3,4].

To address the limitations of conventional concrete, engineered cementitious composites (ECCs) have emerged as an innovative and high-performance alternative within the domain of advanced cement-based materials. ECCs are designed to mitigate the brittleness inherent in traditional concrete, achieving tensile strain capacities several orders of magnitude higher through a distinctive strain-hardening mechanism accompanied by distributed micro-cracking [5,6,7,8]. The superior mechanical performance of ECCs is primarily attributed to the integration of synthetic fibers, such as polyethylene (PE) or polyvinyl alcohol (PVA), which enhance their strain-hardening behavior and crack-bridging capacity. By tailoring the fiber type and content, ECCs can achieve optimized durability, mechanical strength, and thermal stability for specific engineering applications [9,10,11,12,13,14].

While ECCs offer notable advantages, their performance in aggressive environments (such as those involving high alkalinity, freeze–thaw cycles, or elevated temperatures) remains a challenge, potentially compromising their durability and limiting their long-term reliability [15,16,17,18]. Moreover, ECCs’ inherent tensile strength and flexural rigidity may fall short in high-stress applications, necessitating advanced reinforcement strategies. The incorporation of high-strength steel-strand mesh (HSSM) has proven effective in addressing these limitations, significantly enhancing ECCs’ load-bearing capacity and reducing deformation under extreme conditions, thereby extending their applicability to demanding engineering scenarios [18,19,20,21]. The integration of high-strength steel strands with ECCs enhances the materials’ tensile strength and flexural rigidity while maintaining their strain-hardening and micro-cracking characteristics [21,22,23,24,25]. This combination improves load-bearing capacity, crack resistance, and durability, making HSSM-ECCs particularly suitable for high-stress and long-span applications where conventional reinforcements fall short. Research on the behavior of HSSM-reinforced ECCs under varying loading and environmental conditions remains limited [25,26,27,28]. Critical parameters such as reinforcement ratios, slab geometries, and fiber–steel interaction mechanisms need systematic investigation to optimize structural performance [28,29,30,31]. The absence of robust theoretical frameworks and advanced finite element models limits the ability to predict performance with accuracy and hampers their application in large-scale engineering projects [31,32,33,34,35].

This study focuses on addressing the limitations of current research by systematically investigating the flexural behavior of HSSM-reinforced ECC slabs. Utilizing finite element analysis (FEA) and theoretical modeling, this study aims to elucidate the mechanisms governing the composite material’s response to flexural stresses under varying design conditions. Key parameters, including reinforcement ratios, slab geometries, and material properties, will be analyzed to quantify their influence on structural performance. The primary objective is to develop a predictive model for load-bearing capacity, providing critical insights to optimize the design and practical application of HSSM-reinforced ECCs in engineering scenarios.

## 2. Finite Element Analysis (FEA)

### 2.1. Material and Constitutive Model

The bending performance of the HSSM-reinforced ECC slab was evaluated using ABAQUS (2020 version) finite element software. The Concrete Damaged Plasticity (CDP) model, which incorporates elastic damage theory and plasticity principles under tension and compression, was used to simulate the nonlinear behavior of ECCs, with model parameters set as shown in Table 1. To ensure precise numerical analysis, the actual stress–strain relationship of ECCs was employed in the model. The experimental data provided nominal stress and strain values, which were converted into true stress and strain using the following transformation: A quasi-static nonlinear analysis was performed, with a time step selected based on stability and convergence requirements. A mesh size of 5 mm was chosen after considering geometry and computational resources. Mesh sensitivity analyses were conducted by comparing different mesh sizes (e.g., 5 mm, 10 mm, and 15 mm) to balance computational efficiency and result accuracy.

(1) $$\left\{\begin{array}{l} \sigma_{\mathrm{true}}=\frac{P}{A}=\frac{Pl}{l_{0}A_{0}}=\sigma_{nom}(1+\varepsilon_{nom})\\ \varepsilon_{\mathrm{true}}=\int_{l_{0}}^{l}\frac{dl}{l}=\ln(1+\varepsilon_{nom}) \end{array}\right.$$

where *P* represents the load applied to the material, *A*_0_ is the original cross-sectional area of the material, *l* is the current length of the material, and *l*_0_ denotes the initial length of the material.

The tensile constitutive behavior of the ECCs is modeled using a bilinear model approach, as described in Equation (2). The compressive constitutive behavior is based on the model proposed in reference [33], with its corresponding equation outlined in Equation (3).

(2) $$\sigma_{e,t}=\left\{\begin{array}{ll} \frac{\sigma_{e,t}}{\varepsilon_{e,\mathrm{tc}}}\varepsilon_{e,t\;} & \left(\varepsilon_{e,t}\leq \varepsilon_{e,\mathrm{tc}}\right)\\ (0.31\frac{\varepsilon_{e,t}}{\varepsilon_{e,\mathrm{tp}}}+0.69)\sigma_{e,\mathrm{tp}\;} & \left(\varepsilon_{e,\mathrm{tc}}\leq \varepsilon_{e,t}\leq \varepsilon_{e,\mathrm{tp}}\right) \end{array}\right.$$

(3) $$\sigma_{e,c}=\left\{\begin{array}{ll} (1.1\frac{\varepsilon_{e,c}}{\varepsilon_{e,\mathrm{cl}}}+0.5{\left(\frac{\varepsilon_{e,c}}{\varepsilon_{e,\mathrm{cl}}}\right)}^{2}-0.6{\left(\frac{\varepsilon_{e,c}}{\varepsilon_{e,\mathrm{cl}}}\right)}^{6})\sigma_{e,\mathrm{cp}\;} & \left(0\leq \frac{\varepsilon_{e,c}}{\varepsilon_{e,\mathrm{cl}}}\leq 1\right)\\ (\frac{0.15{\left(\frac{\varepsilon_{e,c}}{\varepsilon_{e,\mathrm{cl}}}\right)}^{2}}{1-2\frac{\varepsilon_{e,c}}{\varepsilon_{e,\mathrm{cl}}}+1.15{\left(\frac{\varepsilon_{e,c}}{\varepsilon_{e,\mathrm{cl}}}\right)}^{2}})\sigma_{e,\mathrm{cp}\;} & \left(1\leq \frac{\varepsilon_{e,c}}{\varepsilon_{e,\mathrm{cl}}}\right) \end{array}\right.$$

where *σ_e,t_*, *σ_e,tc_*, and *σ_e,tp_* denote the tensile stress, cracking stress, and ultimate tensile stress of the ECCs, respectively; correspondingly, *ε_e_*, *ε_e,tc_*, and *ε_e,tp_* represent the strain, cracking strain, and the strain corresponding to the ultimate tensile stress of the ECCs. Additionally, *σ_e,cp_* refers to the ultimate compressive stress of the ECCs, while *ε_e,cl_* indicates the strain corresponding to the ultimate compressive stress of the ECCs, which is converted to true stress based on the experimental data for accurate modeling.

### 2.2. Model Formulation and Design Parameters

**Figure 1:**
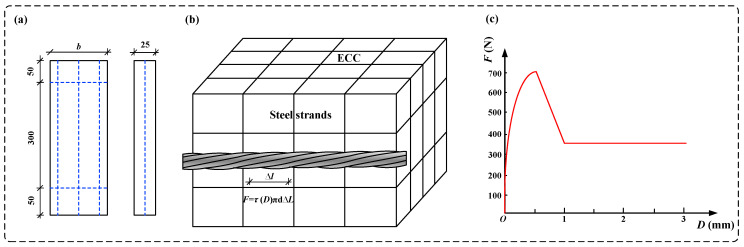
(**a**) The dimensions of the specimens; (**b**) the schematic diagram of the *F*-*D* definition for the spring element; (**c**) the *F*-*D* curve.

The ECC slab was modeled using C3D8R solid elements, while the high-strength steel strands were modeled using T3D2 truss elements. Cushion blocks were placed at the supports and loading points, with a tied interface used to connect them to the ECC slab. The bond–slip behavior between the ECC and the steel strands was modeled through two-node spring elements. This bond–slip interaction was simplified into tangential and normal stresses. Since bond–slip predominantly manifests as shear stress along the length of the steel strands, the normal direction was treated as elastic, with a high stiffness applied to prevent slippage. The *F* (load)–*D* (displacement of the nonlinear spring element) curve describing the bond–slip behavior in the longitudinal tangential direction of the steel strands is shown in Figure 1b, c. The dimensions and reinforcement details of the HSSM-ECC slab bending specimens are shown in Figure 1a, and the design parameters of these specimens are listed in Table 2.

### 2.3. Verification of the Model

**Figure 2:**
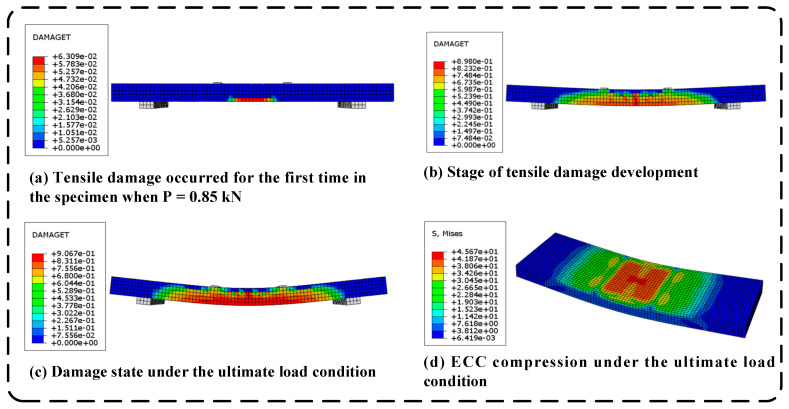
Finite element simulation results of the WC32 specimen.

To validate the accuracy of the finite element model developed, the results of the numerical simulations were compared with the bending performance test results from experiments on the ECC slab/steel strand specimens. The finite element simulation process for specimen WC32 is illustrated in Figure 2. The simulation results indicate that, before reaching the cracking load, the strain in the ECC remained below the cracking strain, and no damage was detected in the tensile zone of the slab. When the load reached 0.85 kN, tensile damage initially appeared in the pure bending zone at the bottom of the slab, indicating the onset of cracking. As the load continued to increase, while still remaining below the ultimate load, the tensile damage in the pure bending zone began to propagate vertically from the bottom of the tensile zone, with the damage values continuing to escalate. The damage became increasingly concentrated in the center of the slab, and diagonal damage emerged in the bending–shear zone, gradually extending outward. When the load reached 4.70 kN, the slab entered the ultimate load state, at which point the damaged area ceased to expand, and the damage values reached their maximum damage factor, signifying that the damage had reached its limit.

**Figure 3:**
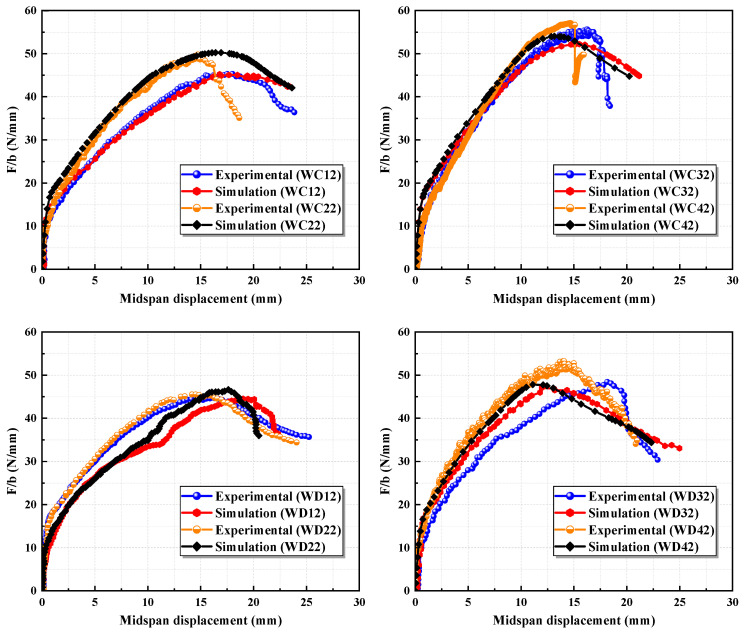
Comparison of the load–midspan displacement curves between the experimental and simulation results.

The comparison of the simulated results with the experimental load–midspan displacement curves for each specimen is presented in Figure 3. Additionally, Table 3 includes the numerical values of the ultimate loads along with their corresponding error values. The simulated ultimate loads closely align with the experimental values, yielding an average error of 4.60% and a maximum error of only 8.99%. Notably, the ascending branch of the finite element simulation curves closely corresponds to the ascending branch of the experimental curves, further validating the accuracy of the established model.

### 2.4. Parametric Simulation Analysis of the Bending Performance of HSSM-ECC Slabs

#### 2.4.1. Longitudinal Steel-Strand Reinforcement Ratio

Using the established model parameters, six sets of specimens were designed to examine the impact of the longitudinal steel-strand reinforcement ratio on the flexural performance of the slabs. The parameters for these specimens are detailed in Table 4. All specimens were uniformly designed with a sectional height of 80 mm. Based on the existing theoretical expression, the maximum reinforcement ratio for this height is 0.57%. The compressive strength of the ECC cube is measured at 44.65 MPa.

**Figure 4:**
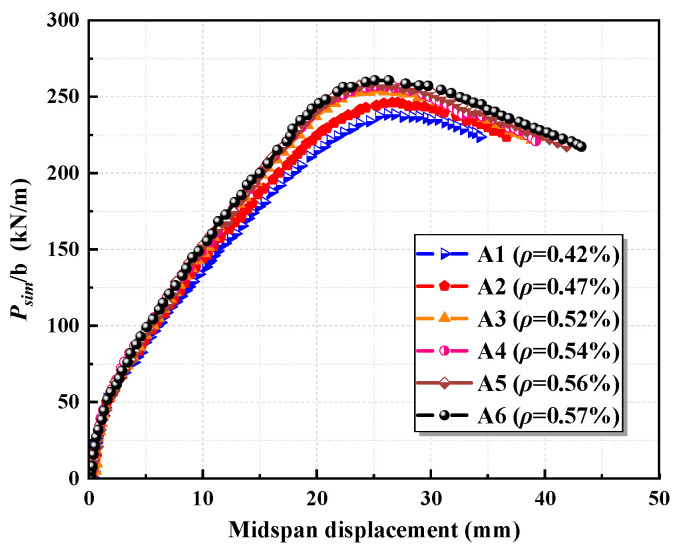
Simulation results with varying longitudinal steel-strand reinforcement ratios.

**Figure 5:**
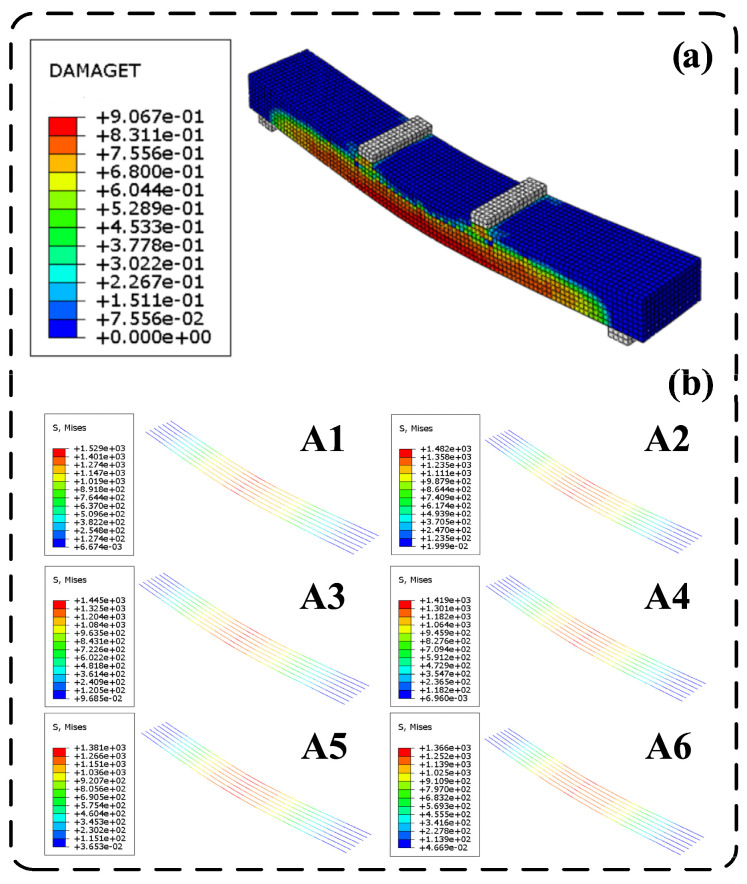
(**a**) Damage factor reaching the limit in the compressed ECC zone; (**b**) stress distribution of longitudinal steel strands at ECC’s ultimate state.

The results from the finite element simulations are illustrated in Figure 4. The load comparison is represented as the load divided by the width (*P_sim_*_/b_). It is observed that, as the longitudinal reinforcement ratio increases, the stiffness of the slab significantly improves, and its load-bearing capacity increases. Meanwhile, the deflection corresponding to the ultimate load progressively decreases. By increasing the longitudinal steel-strand reinforcement ratio, the tensile force within the tension zone within the slab increases for the same curvature. This change causes the resultant force point of the cross-section to shift downward to maintain equilibrium, while the ultimate compressive strain of the ECC remains constant, resulting in reduced deflection at the ultimate load. During the finite element simulations, the ultimate compressive strength of the ECC can be determined by identifying when the maximum damage factor in the compressed region of the ECC reaches its limit, as illustrated in Figure 5. The maximum reinforcement ratio of the slab is confirmed by assessing whether the stress state of the longitudinal steel strands attains the nominal yield state. In the finite element simulation of Group A slabs, the stress of the steel strands was recorded at the moment the ECC reached its ultimate compressive strength, as depicted in Figure 5.

In specimens A1 through A6, as the reinforcement ratio of the slab increases, the stress in the steel strands at the point when the ECC attains its ultimate compressive stress is measured at 1529 MPa, 1482 MPa, 1445 MPa, 1419 MPa, 1381 MPa, and 1366 MPa, respectively. This indicates a continuous decrease in the stress of the steel strands. As the ECC reaches its ultimate compressive stress, the tensile force increases with the reinforcement ratio, causing the neutral axis to shift downward, and the strain and stress in the steel strands decrease. The steel strands in specimen A6 can be considered to have approximately reached the nominal yield stress of 1350 MPa.

#### 2.4.2. Cross-Sectional Geometric Parameters

Simulation analyses were performed on slabs with varying geometric dimensions under three different scenarios: (1) maintaining a constant width while varying the height, (2) keeping the height constant while adjusting the width, and (3) preserving a constant cross-sectional area by simultaneously altering both the width and height. As detailed in Table 5, steel strands with diameters of 3.2 mm and 4.5 mm were employed, with the longitudinal reinforcement ratio being controlled between its minimum and maximum limits. The compressive strength of the ECC was established at 44.65 MPa.

**Figure 6:**
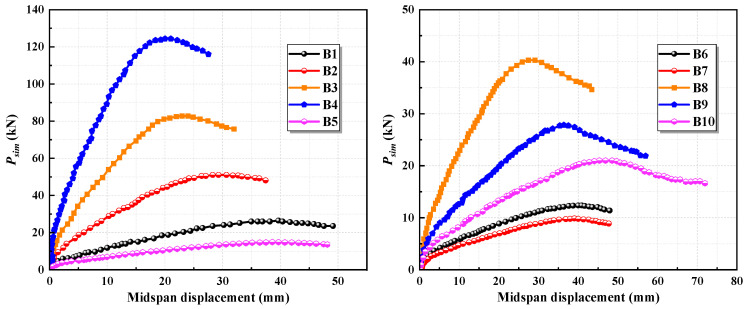
Simulation results with varying geometric parameters of the cross-section.

The results of the finite element simulations are presented in Figure 6. It is observed that an increase in cross-sectional height significantly enhances the flexural stiffness and load-bearing capacity of the specimen while effectively managing the deformation at the ultimate load. Meanwhile, the results demonstrate that cross-sectional height is a key factor influencing the ductility of the high-strength steel-strand mesh/ECC slab, whereas the ductility of the slab does not appear to be affected by changes in its cross-sectional width.

#### 2.4.3. Parameters of ECCs

Assuming that the material properties of ECCs can vary independently without impacting other performance parameters and ensuring that the specimens experience appropriate reinforcement failure, several factors are considered: (1) the tensile stress exponent is fixed at 1.5, while increasing the ECCs’ cracking strength and ultimate tensile strength; (2) the cracking strength remains constant, but the ultimate tensile strength is varied by adjusting the tensile stress exponent (Equation (4)) within a range of 1.3 to 1.6; (3) the compressive strength of the ECCs and other related parameters are modified. The cross-sectional dimensions for the simulations are uniformly established at 120 mm × 100 mm. The parameters for the simulated specimens are outlined in Table 6.

(4) $$\sigma_{e,\mathrm{tp}}/\sigma_{e,\mathrm{tc}}\geq 1.3$$

where *σ_e,tp_* represents the ultimate tensile strength and *σ_e,tc_* represents the material’s cracking strength.

**Figure 7:**
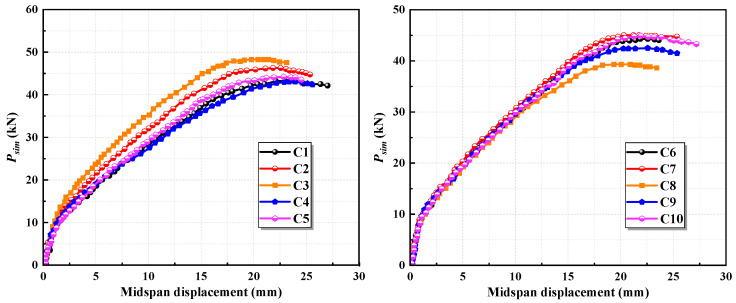
Simulation results with varying ECC parameters.

The simulation outcomes for specimens with varying ECC parameters are illustrated in Figure 7. As seen in the figure, increasing the ECC’s cracking strength and ultimate tensile strength significantly enhances the load-bearing capacity of the slab. However, this increase is accompanied by a decrease in the midspan deflection at the ultimate load, along with a reduction in ductility. When the ECC’s cracking strength remains unchanged and the tensile stress exponent is varied between 1.3 and 1.6, modifications to the ultimate tensile strength yield only minor changes in the load-bearing capacity of the slab, but the variations are negligible, and the load–midspan displacement curves largely overlap. Increasing the ECC’s compressive strength notably improves the load-bearing capacity of the slab while also contributing to improvements in stiffness and ductility.

#### 2.4.4. Sensitivity Analysis

The results indicated that the longitudinal reinforcement ratio had the most significant impact on the slab’s stiffness and load-bearing capacity. A higher reinforcement ratio led to improved load-bearing performance and reduced deflection at ultimate load. Additionally, changes in the ECC’s tensile and compressive strengths also influenced the overall performance, with increases in the ECC’s tensile strength improving load capacity, while higher compressive strength contributed to enhanced stiffness and ductility.

## 3. Theoretical Analysis of Flexural Load-Bearing Capacity Calculation for HSSM-ECC Slabs

### 3.1. Calculating the Load-Bearing Capacity

The calculation model for the flexural load-bearing capacity of HSSM-ECC slabs is based on three key assumptions: (1) Plane-section assumption: the distribution of strain within high-strength steel-strand mesh/ECC slabs under bending adheres to the plane-section assumption. (2) Deformation compatibility between the steel strands and the ECC: throughout the bending process, no relative slip is considered between the ECC and the high-strength steel strands, ensuring deformation compatibility. (3) An ECC in the tension zone remains active after cracking: After an ECC in the tension zone cracks, it is assumed to continue carrying tensile forces. This contribution of the ECC in the tension zone must be considered throughout the entire load-bearing process.

The stress–strain relationship curve of high-strength steel strands adopts a tri-linear model. This can be mathematically expressed as below:

(5) $$\sigma_{s}=\left\{\begin{array}{ll} E_{s1}\varepsilon_{s\;} & \left(0\leq \varepsilon_{s}\leq \varepsilon_{\mathrm{sy}}\right)\\ \sigma_{\mathrm{sy}}+E_{s2}(\varepsilon_{s}-\varepsilon_{sy}) & \left(\varepsilon_{\mathrm{sy}}\leq \varepsilon_{s}\leq \varepsilon_{s2}\right)\\ \sigma_{s2}+E_{s3}(\varepsilon_{s}-\varepsilon_{s2}) & \left(\varepsilon_{s2}\leq \varepsilon_{s}\leq \varepsilon_{su}\right) \end{array}\right.$$

where *E_s_*_1,_ *E_s_*_2_, and *E_s_*_3_ correspond to the deformation moduli of the high-strength steel strands in each of the three stages, respectively. *σ_sy_* is the stress at the end of the first stage, approximately 85% of the ultimate tensile stress, which establishes the nominal yield strength of the steel strands. The corresponding strain at the end of the first stage, *ε_sy_*, is approximately 40% of the ultimate tensile strain. *σ_s_*_2_ is the stress at the end of the second stage, approximately 95% of the ultimate tensile stress, with the corresponding strain *ε_s_*_2_ being approximately 60% of the ultimate tensile strain. *σ_su_* is the ultimate tensile stress of the high-strength steel strands, and *ε_su_* is the ultimate tensile strain.

**Figure 8:**
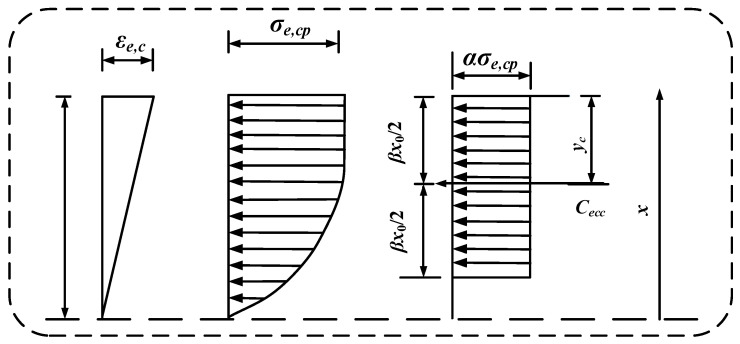
Equivalent rectangular stress distribution diagram for compression zone analysis.

The actual stress distribution within the ECC in the compression zone of the HSSM-ECC slab is simplified into an equivalent rectangular stress distribution, illustrated in Figure 8 (where *C_ecc_* represents the resultant compressive force and *y_c_* is the distance from the point of resultant force to the upper edge of the cross-section).

Accordingly, Equations (6) and (7) can be derived as follows:

(6) $$C_{ecc}=\int_{0}^{x_{0}}\sigma_{e,c}(x)bdx$$

(7) $$y_{c}=x_{0}-\frac{\int_{0}^{x_{0}}\sigma_{e,c}(x)bxdx}{\int_{0}^{x_{0}}\sigma_{e,c}(x)bdx}$$

Based on the principle that the equivalent stress and the actual stress have the same magnitude and point of action, the following can be derived:

(8) $$\left\{\begin{array}{l} \frac{1}{2}\beta x_{0}=y_{c}\\ C_{ecc}=\beta x_{0}\alpha \sigma_{e,cp}b\; \end{array}\right.$$

Substituting Equations (6) and (7) into Equation (8) yields the equivalent stress parameter *α* and the equivalent height parameter *β* for the compression zone.

(9) $$\alpha =\left\{\begin{array}{cc} \frac{\frac{11}{20}\frac{\varepsilon_{e,c}}{\varepsilon_{e,cl}}+\frac{1}{12}{\left(\frac{\varepsilon_{e,c}}{\varepsilon_{e,cl}}\right)}^{5}-\frac{3}{35}{\left(\frac{\varepsilon_{e,c}}{\varepsilon_{e,cl}}\right)}^{6}}{\beta } & \left(\;0\leq \varepsilon_{e,c}<\varepsilon_{e,cl}\right)\\ \frac{1-\frac{19}{42}\frac{\varepsilon_{e,c}}{\varepsilon_{e,cl}}}{\beta } & (\;\varepsilon_{e,cl}\leq \varepsilon_{e,c}\leq \varepsilon_{e,cp})\; \end{array}\right.$$

(10) $$\beta =\left\{\begin{array}{ll} \frac{154\varepsilon_{e,c}{\varepsilon_{e,cl}}^{5}+10{\varepsilon_{e,c}}^{5}\varepsilon_{e,cl}-9{\varepsilon_{e,c}}^{6}}{231\varepsilon_{e,c}{\varepsilon_{e,cl}}^{5}+35{\varepsilon_{e,c}}^{5}\varepsilon_{e,cl}-36{\varepsilon_{e,c}}^{6}} & \;0\leq \varepsilon_{e,c}<\varepsilon_{e,cl}\\ 2-{\frac{84-23\left(\frac{\varepsilon_{e,cl}}{\varepsilon_{e,c}}\right)}{84-38\frac{\varepsilon_{e,cl}}{\varepsilon_{e,c}}}}^{2} & \varepsilon_{e,cl}\leq \varepsilon_{e,c}\leq \varepsilon_{e,cp} \end{array}\right.$$

**Figure 9:**
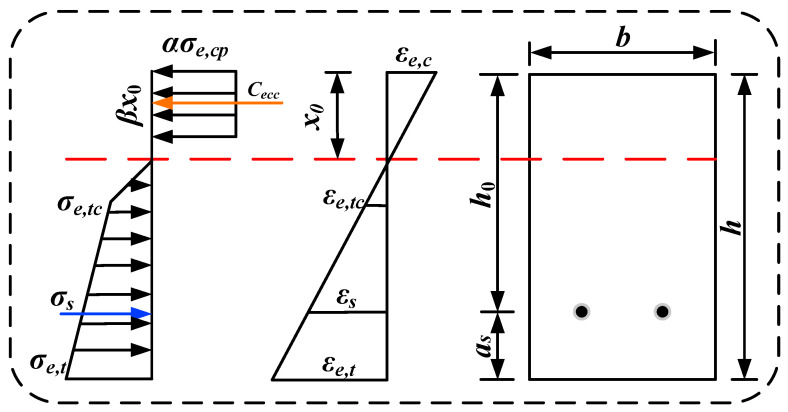
Stress–strain distribution diagram of the cross-section at the moment of ECC crushing failure in the compression zone.

During the calculation of the flexural load-bearing capacity of HSSM-ECC slabs, it is assumed that the ECC in the tension zone remains active. The stress–strain distribution throughout the cross-section is illustrated in Figure 9. In this figure, *b* is the width of the specimen; *h* is the height of the specimen; *x*_0_ is the height of the ECC compression zone; *a_s_* is the steel-strand cover thickness; and *σ_e,tc_*, *ε_e,tc_*, and *ε_e,t_* are the ECC’s cracking stress, cracking strain, and tensile strain, respectively. Meanwhile, *σ_e,cp_* and *ε_e,c_* represent the ultimate compressive stress and compressive strain of the ECC, respectively, while *σ_s_* and *ε_s_* denote the stress and strain of the high-strength steel strands.

By applying the principles of force and moment equilibrium, the ultimate load-bearing capacity can be calculated using the following expression:

(11) $$\left\{\begin{array}{l} \alpha \beta \sigma_{e,cp}bx_{0}=E_{s1}\frac{h-x_{0}-a_{s}}{x_{0}}\varepsilon_{e,cp}A_{s}+\frac{1}{2}\sigma_{e,tc}b\frac{\varepsilon_{e,tc}}{\varepsilon_{e,cp}}x_{0}+\\ \frac{1}{2}\left[(0.31\frac{h-x_{0}}{x_{0}}\frac{\varepsilon_{e,cp}}{\varepsilon_{e,tp}}+0.69)\sigma_{e,tp}+\sigma_{tc}\right]b(h-x_{0}-\frac{\varepsilon_{e,tc}}{\varepsilon_{e,cp}}x_{0})\\ M_{u}=E_{s1}\varepsilon_{s}A_{s}(h-a_{s}-\frac{1}{2}\beta x_{0})+\frac{1}{2}\sigma_{e,tc}b\frac{\varepsilon_{e,tc}}{\varepsilon_{e,cp}}x_{0}(x_{0}-\frac{1}{2}\beta x_{0}+\frac{2}{3}\frac{\varepsilon_{e,tc}}{\varepsilon_{e,cp}}x_{0})\\ +\sigma_{e,tc}b(h-x_{0}-\frac{\varepsilon_{e,tc}}{\varepsilon_{e,cp}}x_{0})\left[h-\frac{1}{2}(h-x_{0}-\frac{\varepsilon_{e,tc}}{\varepsilon_{e,cp}}x_{0})-\frac{1}{2}\beta x_{0}\right]+\\ \frac{1}{2}\left[(m\frac{\varepsilon_{e,t}}{\varepsilon_{e,tc}}+1-m)\sigma_{e,tp}-\sigma_{e,tc}\right]b(h-x_{0}-\frac{\varepsilon_{e,tc}}{\varepsilon_{e,cp}}x_{0})\left[h-\frac{1}{3}(h-x_{0}-\frac{\varepsilon_{e,tc}}{\varepsilon_{e,cp}}x_{0})-\frac{1}{2}\beta x_{0}\right] \end{array}\right.$$

### 3.2. Simplified Analysis of the Flexural Load-Bearing Capacity Calculation Formula

**Figure 10:**
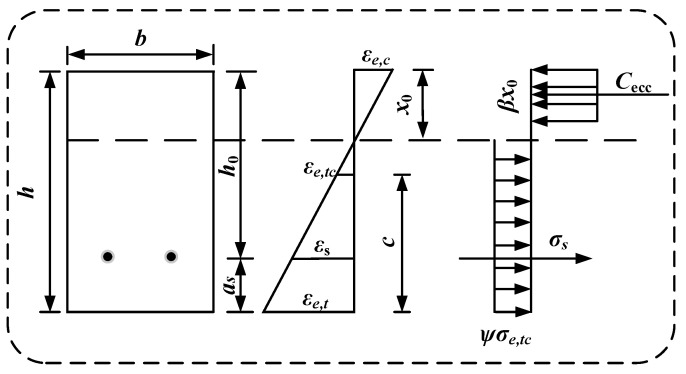
Simplified diagram for the calculation of load-bearing capacity of the cross-section.

In the theoretical calculations, it is assumed that the constitutive models for the high-strength steel strands and the compressive behavior of the ECC remain unchanged. The original bilinear tensile model of ECC is replaced by a modified ideal rigid–plastic model. The simplified stress distribution diagram, which illustrates the flexural load-bearing capacity across the cross-section of the HSSM-ECC slab, is presented in Figure 10.

Using force and moment equilibrium principles, the derivation results in Equation (12):

(12) $$\left\{\begin{array}{l} \alpha \beta \sigma_{e,cp}x_{0}b=\sigma_{s}A_{s}+\psi \sigma_{e,tc}b(h-x_{0})\;\\ M=\sigma_{s}A_{s}(h-a_{s}-\frac{1}{2}\beta x_{0})+\frac{1}{2}\psi \sigma_{e,tc}b(h-x_{0})\;\left[h+(1-\beta )x_{0}\right] \end{array}\right.$$

In the actual experiments, when the specimen failed, the high-strength steel strands did not reach their nominal yield stress and remained in the elastic stage. The strain experienced by the steel strands at the ultimate failure state is described by Equation (13).

(13) $$\varepsilon_{s}=\frac{h-x_{0}-a_{s}}{x_{0}}\varepsilon_{e,cp}$$

Further, the stress expression for the steel strands can be derived as follows:

(14) $$\sigma_{s}=E_{sl}\frac{h-x_{0}-a_{s}}{x_{0}}\varepsilon_{e,cp}$$

By substituting Equation (14) into Equation (13), the formula for calculating the ultimate load-bearing capacity of the flexural specimen at its ultimate state can be obtained using the following expression:

(15) $$\left\{\begin{array}{l} \alpha \beta \sigma_{e,cp}x_{0}b=E_{s1}\frac{h-x_{0}-a_{s}}{x_{0}}\varepsilon_{e,cp}A_{s}+\psi \sigma_{e,tc}b(h-x_{0})\;\\ M=E_{s1}\frac{h-x_{0}-a_{s}}{x_{0}}\varepsilon_{e,cp}A_{s}(h-a_{s}-\frac{1}{2}\beta x_{0})+\frac{1}{2}\psi \sigma_{e,tc}b(h-x_{0})[h+(1-\beta )x_{0}] \end{array}\right.$$

In this study, the value of *ψ* is set to 1.25. The parameters used are as follows: *b* represents the width of the cross-section, and *h* denotes the height of the cross-section. *σ_e,cp_* is the compressive strength of the ECC, while *σ_e,tc_* corresponds to the cracking stress of the ECC. *σ_s_* represents the stress in the high-strength steel strands, and for conditions involving appropriate reinforcement failure, *σ_s_* is taken as *σ_sy_*, which is the nominal yield strength. Additionally, *a_s_* refers to the distance between the high-strength steel strands and the bottom of the cross-section, and *A_s_* stands for the area of the longitudinal high-strength steel strands.

## 4. Verification of the Validity of the Load-Bearing Capacity Calculation Formula

### 4.1. Validation of Specimens Introduced in Section 2.4.1

Table 7 compares the results from the numerical simulations and theoretical calculations for specimens with varying reinforcement ratios. In this comparison, *P_sim_* represents the numerical values derived from finite element simulations, *P*_1_ represents the results calculated using the precise formula in Equation (11), and *P*_2_ represents the results calculated using the simplified formula proposed in Equation (15) in this paper. The ratio of *P*_1_ to *P_sim_* has an average value of 1.018, with a standard deviation of 0.018 and a coefficient of variation of 0.018. Similarly, the ratio of *P*_2_ to *P_sim_* shows an average value of 1.020, with a slightly higher standard deviation of 0.04 and a coefficient of variation of 0.039. This indicates that while the simplified formula introduces marginally more error compared to the precise formula, the error remains within an acceptable range.

Therefore, when the longitudinal steel-strand reinforcement ratio remains below the maximum allowable reinforcement ratio, the calculation formula proposed in this study provides highly accurate and applicable predictions of the load-bearing capacity.

### 4.2. Validation of Specimens Introduced in Section 2.4.2

Table 8 presents a comparison between the finite element simulation results and the theoretical calculations for the specimens discussed in Section 2.4.2. The ratio of *P*_1_ to *P_sim_* has an average value of 0.982, with a standard deviation of 0.013 and a coefficient of variation of 0.013. The ratio of *P*_2_ to *P_sim_* has an average value of 0.990, with a standard deviation of 0.029 and a coefficient of variation of 0.029. These results indicate that both the simplified and the precise expressions provide highly accurate predictions of the ultimate load-bearing capacity for slabs reinforced with steel strands of varying diameters.

### 4.3. Validation of Specimens Introduced in Section 2.4.3

As presented in Table 9, the comparison between the numerical simulation results and theoretical calculations shows that the ratio of *P*_1_ to *P_sim_* has an average value of 1.023, with a standard deviation of 0.015 and a coefficient of variation of 0.015. The ratio of *P*_2_ to *P_sim_* has an average value of 0.982, with a standard deviation of 0.022 and a coefficient of variation of 0.022.

These results confirm that both the precise and simplified formulas provide highly accurate predictions of the ultimate load-bearing capacity of HSSM slabs. The use of the simplified formula does not affect its practical application for calculating the ultimate load-bearing capacity of such slabs in engineering projects.

## 5. Conclusions

1. In this study, finite element analysis was employed to simulate the flexural performance of HSSM-ECC slabs. The findings reveal that as the longitudinal reinforcement ratio of the steel strands increases, the flexural stiffness of the slabs significantly improves, leading to an increase in load-bearing capacity. Additionally, the deflection at the ultimate load decreases, and the post-ultimate load descent phase becomes longer, indicating enhanced performance after reaching the ultimate load.
2. Increasing the ECC’s cracking strength and ultimate tensile strength significantly enhances the load-bearing capacity and stiffness of HSSM-ECC slabs. However, these gains come with reduced ductility and lower deflection at the ultimate load. When the ECC’s compressive strength is increased, the load-bearing capacity also improves, though to a lesser degree. At the same time, both the stiffness and ductility of the slab see noticeable improvements.
3. The improved ideal rigid–plastic tensile model for ECCs was developed through a comparison with existing experimental data to optimize parameter selection. Subsequently, a simplified calculation formula for the flexural load-bearing capacity of HSSM-ECC slabs was formulated. The comparison between the calculated outcomes and the experimental data demonstrated strong alignment, verifying the rationality and accuracy of the proposed calculation method.

The simplified interaction models between steel mesh and ECCs in this study could be further refined. Future research should explore more detailed models and validate the findings with large-scale experiments, including the long-term durability of HSSM-ECC slabs under varying environmental conditions.

## Figures and Tables

**Table 1:** Definition of plasticity parameters for ECCs.

Dilation Angle	Eccentricity	Ratio of Tensile to Compressive Strength in Biaxial Stress	Plastic Potential Eccentricity	Viscosity Parameter
30	0.1	1.16	0.6667	0.0005

**Table 2:** Design parameters of the specimens.

Specimen ID	Specimen Width b/mm	Steel Strand Spacing/mm	Steel Strand Diameter/mm	Number of Steel Strands/n	Reinforcement Ratio of Steel Strands/%	Compressive Strength of ECC
WC12	130	50	2.4	3	0.0026	44.65
WC22	110	40	2.4	3	0.0031	44.65
WC32	90	30	2.4	3	0.0037	44.65
WC42	70	20	2.4	3	0.0048	44.65
WD12	130	50	2.4	3	0.0026	36.05
WD12	110	40	2.4	3	0.0031	36.05
WD12	90	30	2.4	3	0.0037	36.05
WD12	70	20	2.4	3	0.0048	36.05

**Table 3:** Comparison of the relative ultimate load (ultimate load/slab width).

Specimen ID	Ultimate Load F/b (N/mm)		Standard Deviation
	Experimental	Simulation	
WC12	45.99	45.12	1.89%
WC22	49.45	50.25	−1.61%
WC32	56.46	52.23	7.49%
WC42	58.62	54.01	7.86%
WD12	43.81	44.75	−2.15%
WD22	50.00	45.68	8.64%
WD32	49.79	46.95	5.70%
WD42	52.46	47.74	8.99%
Mean standard deviation	4.60%		

**Table 4:** Specimen parameters with varying steel-strand reinforcement ratios.

Specimen ID	Cross-Sectional Dimensions		Steel Strand Diameter (Number of Strands)	Reinforcement Ratio
	Width b/mm	Height h/mm		
A1	45.99	45.12	4.5 mm (7)	0.42%
A2	49.45	50.25	4.5 mm (7)	0.47%
A3	56.46	52.23	4.5 mm (7)	0.52%
A4	58.62	54.01	4.5 mm (7)	0.54%
A5	43.81	44.75	4.5 mm (7)	0.56%
A6	52.46	47.74	4.5 mm (7)	0.57%

**Table 5:** Varying geometric parameters of the cross-section.

Specimen ID	Cross-Sectional Dimensions		Steel Strand Diameter (Number of Strands)	Reinforcement Ratio
	Width b/mm	Height h/mm		
B1	200	60	4.5 mm (6)	0.48%
B2	200	80	4.5 mm (8)	0.48%
B3	200	100	4.5 mm (10)	0.48%
B4	200	120	4.5 mm (12)	0.48%
B5	80	60	3.2 mm (4)	0.41%
B6	100	60	3.2 mm (5)	0.41%
B7	120	60	3.2 mm (6)	0.41%
B8	150	80	4.5 mm (7)	0.56%
B9	200	60	4.5 mm (7)	0.56%
B10	240	50	4.5 mm (7)	0.56%

**Table 6:** Varying parameters of ECCs.

Specimen ID	ECC Parameters				Steel Strand Diameter (Number of Strands)	Reinforcement Ratio
	Cracking Strength/MPa	Ultimate Strength/MPa	Ultimate Tensile Strain/%	Compressive Strength		
C1	2.0	3.0	0.0279	44.65	4.5 mm (5)	0.40%
C2	3.0	4.5	0.0279	44.65	4.5 mm (5)	0.40%
C3	4.0	6.0	0.0279	44.65	4.5 mm (5)	0.40%
C4	2.45	3.18	0.0279	44.65	4.5 mm (5)	0.40%
C5	2.45	3.43	0.0279	44.65	4.5 mm (5)	0.40%
C6	2.45	3.68	0.0279	44.65	4.5 mm (5)	0.40%
C7	2.45	3.92	0.0279	44.65	4.5 mm (5)	0.40%
C8	2.45	3.53	0.0279	35.00	4.5 mm (5)	0.40%
C9	2.45	3.53	0.0279	40.00	4.5 mm (5)	0.40%
C10	2.45	3.53	0.0279	45.00	4.5 mm (5)	0.40%

**Table 7:** Comparison of simulation results and theoretical calculations for specimens in Section 2.4.1.

Specimen ID	Ultimate Load-Bearing Capacity			*P*_1_/*P_sim_*	*P*_2_/*P_sim_*
	*P_sim_*	*P*_1_	*P*_2_		
A1	47.71	46.97	45.39	0.984	0.952
A2	44.27	44.47	43.84	1.005	0.980
A3	41.02	41.88	42.19	1.021	1.029
A4	40.18	41.21	41.77	1.026	1.039
A5	39.07	40.53	41.34	1.037	1.058
A6	38.76	40.12	41.07	1.035	1.059
Average value				1.018	1.020

**Table 8:** Comparison of simulation results and theoretical calculations for specimens in Section 2.4.2.

Specimen ID	Ultimate Load-Bearing Capacity			*P*_1_/*P_sim_*	*P*_2_/*P_sim_*
	*P_sim_*	*P*_1_	*P*_2_		
B1	26.50	25.68	26.13	0.969	0.986
B2	51.16	50.10	50.00	0.979	0.977
B3	85.45	82.52	81.23	0.966	0.951
B4	125.69	123.00	120.10	0.979	0.956
B5	9.76	9.58	9.63	0.982	0.987
B6	12.32	11.99	11.84	0.973	0.961
B7	14.83	14.37	14.19	0.969	0.957
B8	40.25	40.54	41.60	1.007	1.034
B9	27.79	27.67	28.92	0.996	1.041
B10	21.39	21.31	22.58	0.996	1.056
Average value				0.982	0.990

**Table 9:** Comparison of simulation results and theoretical calculations for specimens in Section 2.4.3.

Specimen ID	Ultimate Load-Bearing Capacity			*P*_1_/*P_sim_*	*P*_2_/*P_sim_*
	*P_sim_*	*P*_1_	*P*_2_		
C1	43.23	44.54	41.48	1.030	0.960
C2	46.12	46.51	44.84	1.008	0.972
C3	48.51	48.47	48.03	0.999	0.990
C4	42.93	44.89	43.02	1.046	1.002
C5	44.14	45.16	43.02	1.023	0.975
C6	44.22	45.42	43.02	1.027	0.973
C7	45.08	45.69	43.02	1.014	0.954
C8	39.28	40.94	40.32	1.042	1.026
C9	42.45	43.26	42.36	1.019	0.998
C10	44.52	45.41	43.63	1.020	0.980
Average value				1.023	0.983

## Data Availability

The original contributions presented in the study are included in the article, further inquiries can be directed to the corresponding authors.
